# {4-Amino-*N*′-[(2-oxidonaphthalen-1-yl)methyl­idene]benzohydrazidato}di­methyl­tin(IV)

**DOI:** 10.1107/S1600536814014652

**Published:** 2014-06-25

**Authors:** Miao Cheng, Yuhong Zhao, Tiantian Dong, Jichun Cui

**Affiliations:** a College of Chemistry and Chemical Engineering, Liaocheng University, Shandong 252059, People’s Republic of China

**Keywords:** crystal structure

## Abstract

In the title compound, [Sn(CH_3_)_2_(C_18_H_13_N_3_O_2_)], the Sn^IV^ ion is coordinated by one N and two O atoms from the tridentate 4-amino-*N*′-[(2-oxidonaphthalen-1-yl)methyl­idene]benzohydrazidate(2−) ligand and two C atoms from methyl ligands in a distorted trigonal–bipyramidal geometry. The dihedral angle between the naphthalene ring system and the benzene ring is 19.2 (2)°. In the crystal, weak N—H⋯O hydrogen bonds link the mol­ecules into zigzag chains along [010].

## Related literature   

For the biological activity of organotin compounds, see: Hong *et al.* (2013 ▶). For a related crystal structure, see: Cui *et al.* (2013 ▶).
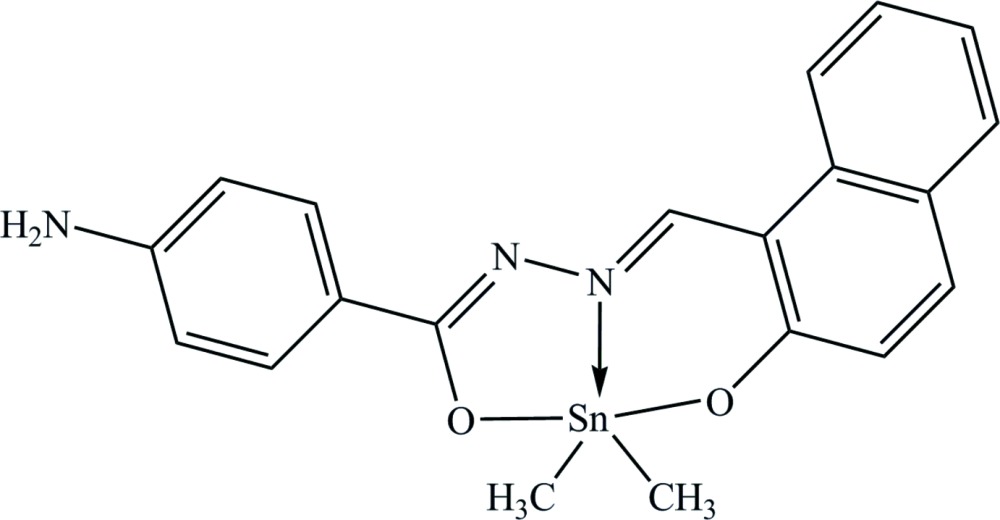


## Experimental   

### 

#### Crystal data  

[Sn(CH_3_)_2_(C_18_H_13_N_3_O_2_)]
*M* *_r_* = 452.07
Orthorhombic, 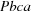

*a* = 8.3545 (7) Å
*b* = 12.9503 (11) Å
*c* = 34.291 (2) Å
*V* = 3710.1 (5) Å^3^
*Z* = 8
Mo *K*α radiation
μ = 1.40 mm^−1^
*T* = 293 K
0.35 × 0.20 × 0.15 mm

#### Data collection  

Bruker SMART 1000 diffractometer
Absorption correction: multi-scan (*SADABS*; Bruker, 2007 ▶) *T*_min_ = 0.641, *T*_max_ = 0.818
8234 measured reflections
3265 independent reflections
2382 reflections with *I* > 2σ(*I*)
*R*_int_ = 0.055

#### Refinement  

*R*[*F* ^2^ > 2σ(*F* ^2^)] = 0.052
*wR*(*F* ^2^) = 0.121
*S* = 1.14
3265 reflections
237 parameters
H-atom parameters constrained
Δρ_max_ = 0.49 e Å^−3^
Δρ_min_ = −1.27 e Å^−3^

### 

Data collection: *SMART* (Bruker, 2007 ▶); cell refinement: *SAINT* (Bruker, 2007 ▶); data reduction: *SAINT*; program(s) used to solve structure: *SHELXS97* (Sheldrick, 2008 ▶); program(s) used to refine structure: *SHELXL97* (Sheldrick, 2008 ▶); molecular graphics: *PLATON* (Spek, 2009 ▶); software used to prepare material for publication: *SHELXTL* (Sheldrick, 2008 ▶).

## Supplementary Material

Crystal structure: contains datablock(s) I. DOI: 10.1107/S1600536814014652/cv5466sup1.cif

Structure factors: contains datablock(s) I. DOI: 10.1107/S1600536814014652/cv5466Isup2.hkl

CCDC reference: 1009501

Additional supporting information:  crystallographic information; 3D view; checkCIF report

## Figures and Tables

**Table 1:** Hydrogen-bond geometry (Å, °)

*D*—H⋯*A*	*D*—H	H⋯*A*	*D*⋯*A*	*D*—H⋯*A*
N3—H3*A*⋯O1^i^	0.86	2.46	3.245 (7)	152

Symmetry code: (i) 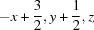.

**Fig. 1.**
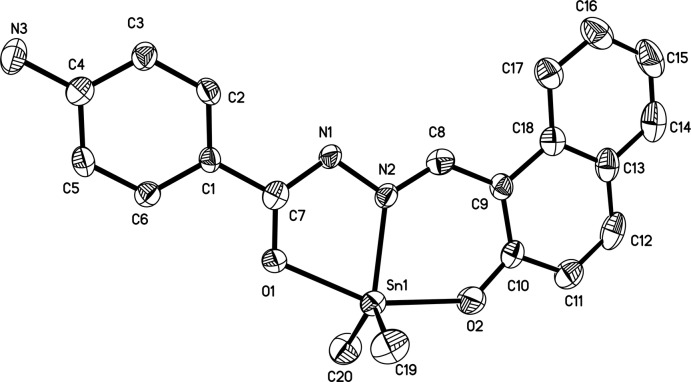
The molecular strucure of (I) showing showing the atomic numbering and 40% probability displacement ellipsoids. H atoms omitted for clarity.

## supplementary crystallographic information

### S1. Comment

The chemistry of organotin(IV) derivatives is a subject of study with growing interest due to their significant antimicrobial properties as well as antitumor activities (Hong *et al.*, 2013). As a part of our ongoing investigations in this field (Cui *et al.*, 2013) we have synthesized the title compound, (I), and present its crystal structure here.

In (I) (Fig. 1), the Sn^IV^ ion has distorted trigonal-bipyramidal coordination geometry, with atoms O1 and O2 in axial positions [O1—Sn1—O3 = 154.81 (18) °] and the atoms C19, C20 and N2 in equatorial positions. This coordination geometry is similar to that observed in the related compound {4-chloro-N'-[(2-oxidonaphthalen-1-ylκO) methylidene]benzohydrazidato-κ^2^N',O}dimethyltin(IV) (Cui *et al.*, 2013).

In the crystal, weak intermolecular N—H···O hydrogen bonds (Table 1) link molecules into zigzag chains in [010].

### S2. Experimental

[4-Azyl-N'-(2-hydroxy-1-naphthaldehyde)benzohydrazide (1 mmol) and sodium ethoxide (2 mmol) were added to the solution of dry methanol(30 ml) and stirred for 10 min. Dimethyltin(IV) dichloride (1 mmol) was then added to the reactor and the reaction mixture was stirred for 4 h. The resulting clear solution was evaporated under vacuum. The product was crystallized from a mixture of dichloromethane/ethanol(1:1) to yield orange blocks of the title compound (yield 86%).

### S3. Refinement

The H atoms were fixed geometrically and treated as riding atoms: N—H = 0.86 Å, with *U*_iso_(H) = 1.2 *U*_eq_(N), and C—H = 0.93 - 0.97 Å, with *U*_iso_(H) = 1.2 or 1.5 *U*_eq_(C)

### Crystal data

**Table:**

[Sn(CH_3_)_2_(C_18_H_13_N_3_O_2_)]	*D*_x_ = 1.619 Mg m^−^^3^
*M**_r_* = 452.07	Mo *K*α radiation, λ = 0.71073 Å
Orthorhombic, *P**b**c**a*	Cell parameters from 1667 reflections
*a* = 8.3545 (7) Å	θ = 2.7–28.4°
*b* = 12.9503 (11) Å	µ = 1.40 mm^−^^1^
*c* = 34.291 (2) Å	*T* = 293 K
*V* = 3710.1 (5) Å^3^	Block, orange
*Z* = 8	0.35 × 0.20 × 0.15 mm
*F*(000) = 1808	

### Data collection

**Table:**

Bruker SMART 1000 diffractometer	3265 independent reflections
Radiation source: fine-focus sealed tube	2382 reflections with *I* > 2σ(*I*)
Graphite monochromator	*R*_int_ = 0.055
phi and ω scans	θ_max_ = 25.0°, θ_min_ = 2.7°
Absorption correction: multi-scan (*SADABS*; Bruker, 2007)	*h* = −9→4
*T*_min_ = 0.641, *T*_max_ = 0.818	*k* = −8→15
8234 measured reflections	*l* = −33→40

### Refinement

**Table:**

Refinement on *F*^2^	Primary atom site location: structure-invariant direct methods
Least-squares matrix: full	Secondary atom site location: difference Fourier map
*R*[*F*^2^ > 2σ(*F*^2^)] = 0.052	Hydrogen site location: inferred from neighbouring sites
*wR*(*F*^2^) = 0.121	H-atom parameters constrained
*S* = 1.14	*w* = 1/[σ^2^(*F*_o_^2^) + (0.034*P*)^2^ + 1.7722*P*] where *P* = (*F*_o_^2^ + 2*F*_c_^2^)/3
3265 reflections	(Δ/σ)_max_ < 0.001
237 parameters	Δρ_max_ = 0.49 e Å^−^^3^
0 restraints	Δρ_min_ = −1.27 e Å^−^^3^

### Fractional atomic coordinates and isotropic or equivalent isotropic displacement parameters (Å^2^)

**Table:**

	*x*	*y*	*z*	*U*_iso_*/*U*_eq_	
Sn1	0.22653 (5)	0.82846 (3)	0.350360 (13)	0.03635 (18)	
C1	0.6096 (7)	1.0398 (4)	0.31885 (17)	0.0282 (13)	
C2	0.6565 (7)	1.1338 (4)	0.33421 (18)	0.0333 (14)	
H2	0.6144	1.1553	0.3580	0.040*	
C3	0.7651 (7)	1.1966 (5)	0.3149 (2)	0.0357 (16)	
H3	0.7940	1.2599	0.3256	0.043*	
C4	0.8311 (7)	1.1654 (5)	0.27934 (19)	0.0346 (14)	
C5	0.7822 (7)	1.0706 (5)	0.26347 (19)	0.0361 (15)	
H5	0.8241	1.0488	0.2397	0.043*	
C6	0.6718 (7)	1.0092 (4)	0.28297 (18)	0.0326 (14)	
H6	0.6391	0.9471	0.2720	0.039*	
C7	0.4895 (7)	0.9742 (5)	0.33911 (19)	0.0337 (15)	
C8	0.3081 (8)	0.9472 (4)	0.4283 (2)	0.0358 (15)	
H8	0.3624	1.0011	0.4404	0.043*	
C9	0.1988 (7)	0.8916 (5)	0.45182 (18)	0.0331 (15)	
C10	0.1039 (7)	0.8101 (4)	0.43669 (19)	0.0365 (15)	
C18	0.1849 (7)	0.9175 (5)	0.4931 (2)	0.0366 (15)	
C11	0.0086 (9)	0.7509 (5)	0.4619 (2)	0.052 (2)	
H11	−0.0513	0.6962	0.4521	0.062*	
C12	0.0036 (9)	0.7732 (6)	0.5005 (2)	0.057 (2)	
H12	−0.0591	0.7321	0.5167	0.069*	
C13	0.0892 (8)	0.8557 (5)	0.5173 (2)	0.0456 (18)	
C14	0.0800 (10)	0.8798 (7)	0.5574 (2)	0.065 (2)	
H14	0.0174	0.8382	0.5734	0.078*	
C15	0.1576 (10)	0.9600 (7)	0.5734 (2)	0.067 (2)	
H15	0.1495	0.9737	0.5999	0.081*	
C16	0.2504 (9)	1.0222 (7)	0.5491 (2)	0.058 (2)	
H16	0.3037	1.0788	0.5596	0.070*	
C17	0.2644 (8)	1.0012 (6)	0.5102 (2)	0.0485 (19)	
H17	0.3282	1.0435	0.4948	0.058*	
C19	0.0383 (9)	0.9080 (5)	0.3224 (2)	0.057 (2)	
H19A	−0.0545	0.9080	0.3389	0.085*	
H19B	0.0132	0.8742	0.2982	0.085*	
H19C	0.0705	0.9778	0.3173	0.085*	
C20	0.3138 (9)	0.6763 (5)	0.3414 (2)	0.056 (2)	
H20A	0.2512	0.6286	0.3564	0.084*	
H20B	0.4237	0.6726	0.3495	0.084*	
H20C	0.3059	0.6590	0.3142	0.084*	
N1	0.4605 (6)	0.9964 (3)	0.37632 (14)	0.0331 (12)	
N2	0.3417 (6)	0.9318 (3)	0.39137 (15)	0.0323 (12)	
N3	0.9344 (7)	1.2295 (4)	0.25953 (17)	0.0493 (15)	
H3A	0.9585	1.2890	0.2690	0.059*	
H3B	0.9749	1.2101	0.2377	0.059*	
O1	0.4219 (5)	0.9000 (3)	0.31939 (12)	0.0393 (11)	
O2	0.0996 (6)	0.7846 (4)	0.39985 (14)	0.0609 (15)	

### Atomic displacement parameters (Å^2^)

**Table:**

	*U*^11^	*U*^22^	*U*^33^	*U*^12^	*U*^13^	*U*^23^
Sn1	0.0369 (3)	0.0357 (3)	0.0364 (3)	−0.00536 (19)	0.0007 (2)	−0.0002 (2)
C1	0.029 (3)	0.032 (3)	0.023 (3)	0.000 (3)	0.002 (3)	0.004 (3)
C2	0.032 (3)	0.040 (3)	0.027 (3)	0.003 (3)	0.004 (3)	−0.005 (3)
C3	0.041 (3)	0.030 (3)	0.036 (4)	−0.002 (3)	0.007 (3)	0.003 (3)
C4	0.024 (3)	0.039 (3)	0.041 (4)	0.004 (3)	−0.002 (3)	0.002 (3)
C5	0.034 (3)	0.048 (4)	0.026 (3)	0.003 (3)	0.004 (3)	0.004 (3)
C6	0.032 (3)	0.033 (3)	0.033 (4)	−0.002 (3)	−0.003 (3)	0.003 (3)
C7	0.026 (3)	0.035 (3)	0.040 (4)	0.007 (3)	−0.002 (3)	0.006 (3)
C8	0.043 (3)	0.027 (3)	0.037 (4)	−0.004 (3)	−0.003 (3)	0.009 (3)
C9	0.036 (3)	0.039 (3)	0.024 (3)	−0.001 (3)	0.001 (3)	0.007 (3)
C10	0.032 (3)	0.039 (3)	0.039 (4)	−0.003 (3)	0.005 (3)	0.007 (3)
C18	0.033 (3)	0.038 (4)	0.038 (4)	0.009 (3)	−0.001 (3)	0.006 (3)
C11	0.054 (4)	0.043 (4)	0.057 (6)	−0.014 (4)	0.008 (4)	0.005 (4)
C12	0.048 (4)	0.064 (5)	0.060 (6)	−0.006 (4)	0.018 (4)	0.026 (4)
C13	0.039 (4)	0.062 (5)	0.036 (4)	0.009 (3)	0.005 (4)	0.013 (4)
C14	0.053 (5)	0.086 (6)	0.055 (6)	0.010 (5)	0.017 (5)	0.018 (5)
C15	0.073 (6)	0.097 (7)	0.032 (4)	0.015 (5)	0.006 (5)	−0.005 (5)
C16	0.059 (5)	0.071 (5)	0.044 (5)	0.014 (4)	−0.010 (4)	−0.013 (4)
C17	0.052 (4)	0.054 (4)	0.039 (4)	0.005 (3)	0.001 (4)	0.000 (4)
C19	0.053 (4)	0.052 (4)	0.066 (5)	0.001 (4)	−0.010 (4)	0.007 (4)
C20	0.055 (4)	0.038 (4)	0.075 (6)	0.002 (4)	0.005 (4)	−0.004 (4)
N1	0.035 (3)	0.041 (3)	0.023 (3)	−0.013 (2)	0.000 (3)	0.005 (2)
N2	0.030 (3)	0.037 (3)	0.030 (3)	−0.007 (2)	0.002 (3)	0.004 (2)
N3	0.050 (3)	0.043 (3)	0.054 (4)	−0.004 (3)	0.020 (3)	0.006 (3)
O1	0.048 (2)	0.037 (2)	0.033 (3)	−0.014 (2)	0.006 (2)	−0.005 (2)
O2	0.075 (4)	0.065 (3)	0.043 (3)	−0.039 (3)	0.012 (3)	−0.001 (3)

### Geometric parameters (Å, º)

**Table:**

Sn1—O2	2.080 (5)	C10—C11	1.403 (9)
Sn1—C19	2.110 (7)	C18—C17	1.401 (9)
Sn1—C20	2.123 (6)	C18—C13	1.402 (9)
Sn1—O1	2.157 (4)	C11—C12	1.356 (10)
Sn1—N2	2.167 (5)	C11—H11	0.9300
C1—C2	1.383 (8)	C12—C13	1.409 (10)
C1—C6	1.393 (8)	C12—H12	0.9300
C1—C7	1.487 (8)	C13—C14	1.412 (10)
C2—C3	1.388 (8)	C14—C15	1.342 (11)
C2—H2	0.9300	C14—H14	0.9300
C3—C4	1.397 (9)	C15—C16	1.394 (11)
C3—H3	0.9300	C15—H15	0.9300
C4—N3	1.377 (8)	C16—C17	1.366 (10)
C4—C5	1.403 (8)	C16—H16	0.9300
C5—C6	1.389 (8)	C17—H17	0.9300
C5—H5	0.9300	C19—H19A	0.9600
C6—H6	0.9300	C19—H19B	0.9600
C7—O1	1.304 (7)	C19—H19C	0.9600
C7—N1	1.330 (8)	C20—H20A	0.9600
C8—N2	1.311 (8)	C20—H20B	0.9600
C8—C9	1.416 (8)	C20—H20C	0.9600
C8—H8	0.9300	N1—N2	1.397 (6)
C9—C10	1.418 (8)	N3—H3A	0.8600
C9—C18	1.458 (9)	N3—H3B	0.8600
C10—O2	1.306 (7)		
			
O2—Sn1—C19	97.1 (3)	C12—C11—C10	120.2 (7)
O2—Sn1—C20	92.3 (3)	C12—C11—H11	119.9
C19—Sn1—C20	130.0 (3)	C10—C11—H11	119.9
O2—Sn1—O1	154.81 (18)	C11—C12—C13	122.9 (7)
C19—Sn1—O1	97.5 (2)	C11—C12—H12	118.5
C20—Sn1—O1	93.9 (2)	C13—C12—H12	118.5
O2—Sn1—N2	82.28 (18)	C18—C13—C12	118.8 (7)
C19—Sn1—N2	108.9 (2)	C18—C13—C14	118.8 (7)
C20—Sn1—N2	121.0 (2)	C12—C13—C14	122.5 (7)
O1—Sn1—N2	73.63 (17)	C15—C14—C13	122.9 (8)
C2—C1—C6	118.8 (5)	C15—C14—H14	118.5
C2—C1—C7	121.0 (5)	C13—C14—H14	118.5
C6—C1—C7	120.1 (5)	C14—C15—C16	118.1 (7)
C1—C2—C3	121.3 (6)	C14—C15—H15	120.9
C1—C2—H2	119.4	C16—C15—H15	120.9
C3—C2—H2	119.4	C17—C16—C15	121.0 (8)
C2—C3—C4	120.3 (6)	C17—C16—H16	119.5
C2—C3—H3	119.8	C15—C16—H16	119.5
C4—C3—H3	119.8	C16—C17—C18	121.6 (8)
N3—C4—C3	120.2 (6)	C16—C17—H17	119.2
N3—C4—C5	121.2 (6)	C18—C17—H17	119.2
C3—C4—C5	118.5 (6)	Sn1—C19—H19A	109.5
C6—C5—C4	120.4 (6)	Sn1—C19—H19B	109.5
C6—C5—H5	119.8	H19A—C19—H19B	109.5
C4—C5—H5	119.8	Sn1—C19—H19C	109.5
C5—C6—C1	120.7 (6)	H19A—C19—H19C	109.5
C5—C6—H6	119.7	H19B—C19—H19C	109.5
C1—C6—H6	119.7	Sn1—C20—H20A	109.5
O1—C7—N1	125.3 (5)	Sn1—C20—H20B	109.5
O1—C7—C1	118.1 (5)	H20A—C20—H20B	109.5
N1—C7—C1	116.6 (5)	Sn1—C20—H20C	109.5
N2—C8—C9	127.7 (6)	H20A—C20—H20C	109.5
N2—C8—H8	116.2	H20B—C20—H20C	109.5
C9—C8—H8	116.2	C7—N1—N2	110.8 (5)
C8—C9—C10	122.0 (6)	C8—N2—N1	114.7 (5)
C8—C9—C18	119.2 (6)	C8—N2—Sn1	128.7 (4)
C10—C9—C18	118.8 (6)	N1—N2—Sn1	116.5 (4)
O2—C10—C11	116.2 (6)	C4—N3—H3A	120.0
O2—C10—C9	123.9 (6)	C4—N3—H3B	120.0
C11—C10—C9	119.9 (6)	H3A—N3—H3B	120.0
C17—C18—C13	117.6 (7)	C7—O1—Sn1	112.9 (4)
C17—C18—C9	123.2 (6)	C10—O2—Sn1	134.9 (4)
C13—C18—C9	119.2 (6)		

### Hydrogen-bond geometry (Å, º)

**Table:**

*D*—H···*A*	*D*—H	H···*A*	*D*···*A*	*D*—H···*A*
N3—H3*A*···O1^i^	0.86	2.46	3.245 (7)	152

Symmetry code: (i) −*x*+3/2, *y*+1/2, *z*.
